# Surgical treatment of metastatic mesenchymal chondrosarcoma to the spine: A case report

**DOI:** 10.1097/MD.0000000000018643

**Published:** 2020-01-31

**Authors:** Shuzhong Liu, Xi Zhou, An Song, Zhen Huo, Yipeng Wang, Yong Liu

**Affiliations:** a Department of Orthopaedic Surgery, Peking Union Medical College Hospital, Peking Union Medical College and Chinese Academy of Medical Sciences,; b Department of Endocrinology, Key Laboratory of Endocrinology, National Health and Family Planning Commission, Peking Union Medical College Hospital, Chinese Academy of Medical Science & Peking Union Medical College,; c Department of Pathology, Peking Union Medical College Hospital, Chinese Academy of Medical Science & Peking Union Medical College, Beijing, China.

**Keywords:** cement augmentation, diagnosis, mesenchymal chondrosarcoma, metastatic spinal chondrosarcoma, recurrence, spine, surgical treatment

## Abstract

**Introduction:**

Metastatic mesenchymal chondrosarcoma of the spine is a highly unusual disease without standard curative managements yet. The objective of this case report is to present a very rare case of metastatic chondrosarcoma to the spine successfully operated by surgical treatment. The management of these unique cases has yet to be well-documented.

**Patient concerns:**

A 34-year-old woman presented with a 4-month history of continuous and progressive back pain and a 1-month history of radiating pain of bilateral lower extremities. The patient, who had been diagnosed of mesenchymal chondrosarcoma of maxillary sinus for 3 years, received surgical treatment of palliative endoscopic-assisted total left maxillary resection via mini Caldwell-Luc approach, and palliative enlarged resection due to the progress of residual lesions, followed by no adjuvant therapy. Multiple lytic, expanding lesions of the spine and paraspinal region with severe epidural spinal cord compression was identified.

**Diagnosis:**

CT, MRI and bone scan of spine showed spinal cord compression secondary to the epidural component of the metastatic lesions. Post-operative pathology confirmed the diagnosis of metastatic spinal mesenchymal chondrosarcomas.

**Interventions:**

The patient underwent posterior spinal canal decompression, resection of T12 and L3 lesions, internal fixation of T11-L5 pedicles, and cement augmentation of T12 and L3.

**Outcomes:**

The patient's neurological deficits improved significantly after the surgery, and the postoperative period was uneventful at the 1-year follow-up visit. There were no complications associated with the spinal surgery during the follow-up period.

**Conclusion:**

Metastatic spinal mesenchymal chondrosarcoma, although rare, should be part of the differential diagnosis when the patient presents with back pain and radiculopathy. We recommend the posterior approach for spinal decompression and total resection of the metastatic chondrosarcoma when the tumor has caused neurological deficits or other severe symptoms. Osteoplasty by cement augmentation is also a good choice for surgical treatment in some patients.

## 1 Introduction

Chondrosarcoma is a malignant tumor which is comprised of transformed cells producing the cartilaginous matrix without tumor osteoid tissue.^[1]^ Its estimated annual incidence is 1 in 200,000 to 500,000 and mesenchymal chondrosarcoma accounts for 0.2% to 0.7% of malignant bone tumors or 3% to 10% of chondrosarcoma.^[1–3]^ Within the whole spine, chondrosarcoma has a predilection for the thoracic spine, but can arise from anywhere along the length from cervical spine to sacrum.^[4,5]^ It typically develops in the vertebral body with extension into the posterior elements, and malignant mesenchymal chondrosarcoma with multiple vertebral metastases causing severe symptoms is extraordinarily rare.^[1–3]^

To the best of our knowledge, this is a rare case of metastatic spinal mesenchymal chondrosarcoma in a woman presenting with radiculopathy, who underwent surgical treatment. In the follow-up period, the patient's conditions improved significantly postoperatively. After reviewing pertinent literatures, we discussed common perioperative considerations in patients with metastatic chondrosarcoma of the spine and management considerations for these unique cases.

## 2 Case report

**Figure 1:**
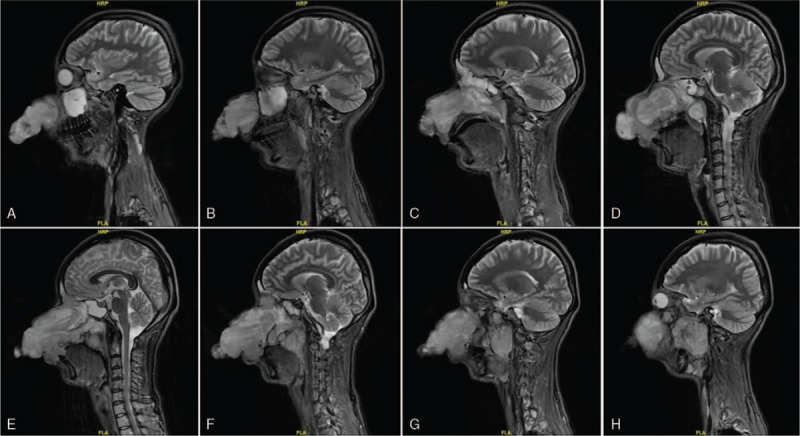
(A–H) Skull sagittal MRI revealed giant occupying lesions with abnormal signals in ethmoid sinus and left infratemporal fossa.

**Figure 2:**
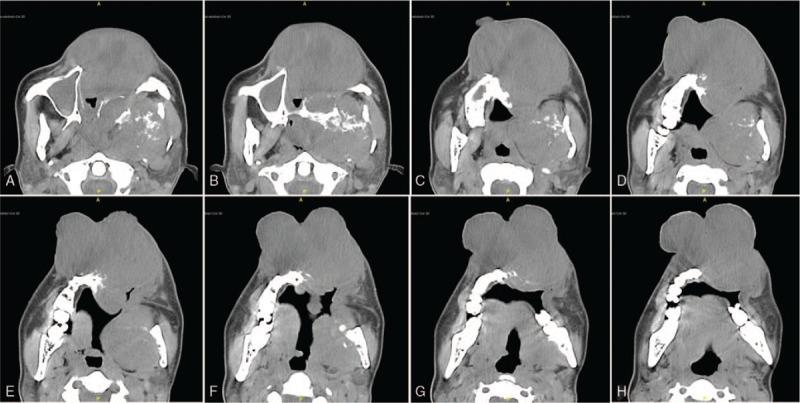
(A–H) Skull transverse CT scan revealed giant occupying lesions with extranasal involvement.

**Figure 3:**
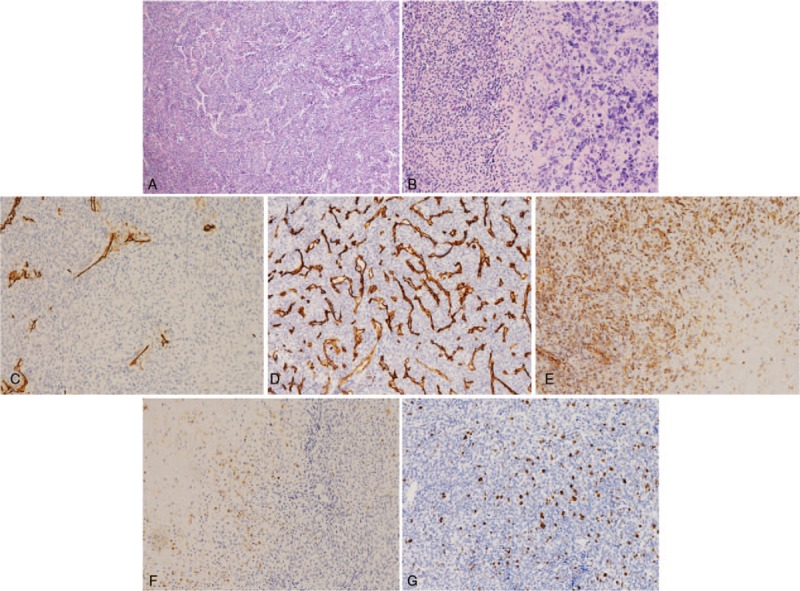
(A–G) The postoperative pathological results confirmed mesenchymal chondrosarcoma after the first and second maxillofacial surgery.

In March of 2016, a 34-year-old woman presented to our hospital, with progressive back pain, and radiating pain of her bilateral lower limbs. In the medical journal of her current illness, the patient stated she had been experiencing a worsening numbness and radiating pain of her bilateral lower limbs for approximately one month, and she had also experienced paroxysmal back pain for approximately four months. The pain in her back can reach 7 to 8 points using visual analogue scale (VAS) and cannot be alleviated with rest and hot compresses. Upon further questioning, she recalled the history of mesenchymal chondrosarcoma of maxillary sinus (Figs. 1A–H and 2A-H). The patient, who had been diagnosed of mesenchymal chondrosarcoma of maxillary sinus for 3 years, received surgical treatments including endoscopic-assisted total left maxillary resection via mini Caldwell-Luc approach, followed by palliative enlarged resection due to the progress of residual lesions, and no adjuvant therapy. The pathological results confirmed mesenchymal chondrosarcoma (Fig. 3A–G). The patient denied experiencing any other constitutional symptoms. No pertinent family history was identified, including hypertension and cancer.

**Figure 4:**
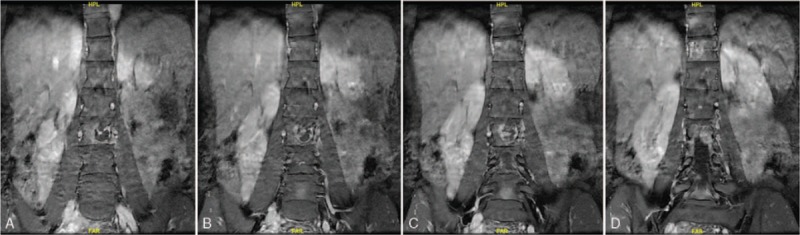
(A–D) Preoperative coronal MRI scan revealing the density of soft tissues and obvious bony destruction in the T12 and L3.

**Figure 5:**
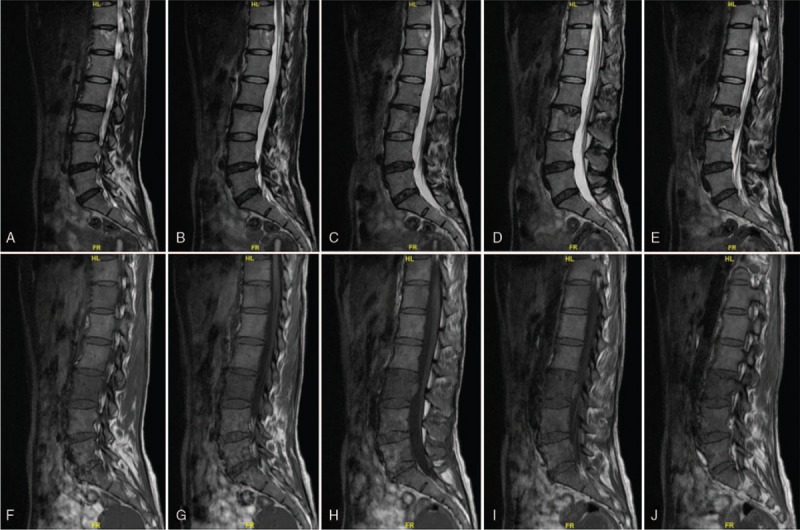
(A–D) Preoperative sagittal MRI scan revealing the density of soft tissues and obvious bony destruction in the T12 and L3, and spinal cord compression caused by thoracic tumor, with increased metastatic marrow infiltration of the vertebra.

**Figure 6:**
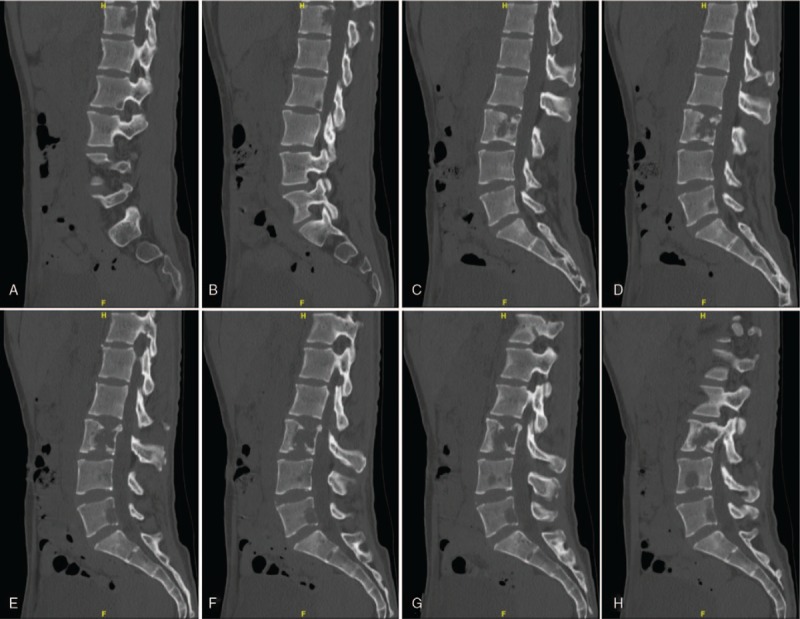
(A–J) Spinal sagittal CT scan revealed obvious bony destruction in the T12 and L3 with intraspinal involvement.

**Figure 7:**
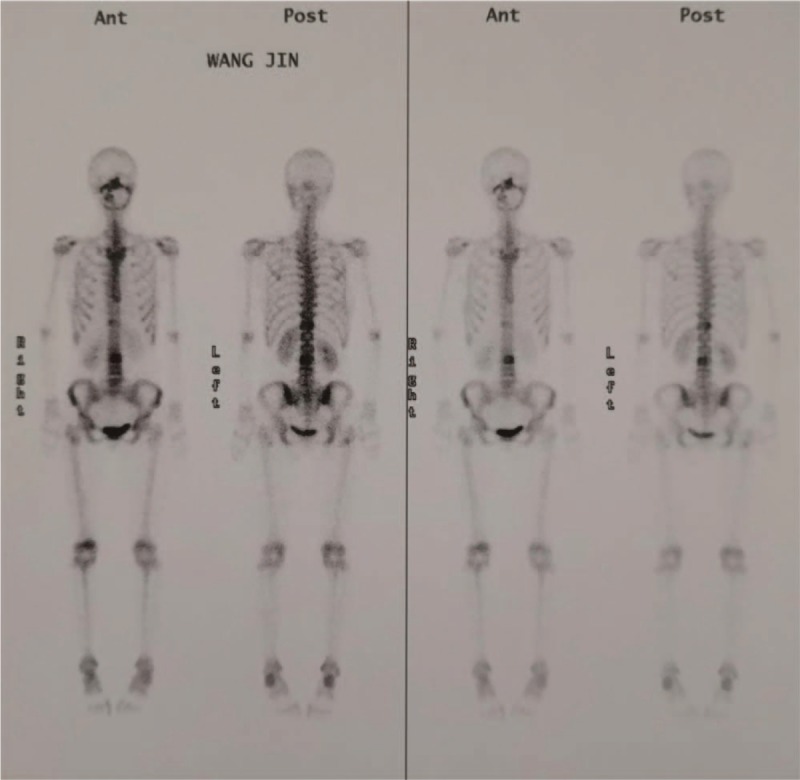
Bone scan revealed high intake in the thoracic spine, with high suspicion of malignant solid tumor.

On physical exam, the patient showed pressure pain and percussion pain at the T12 and L3 lumbar region, decreased sensation to pin-prick and fine-touch of her bilateral lower limbs and exhibited an 5-/5 strength in her bilateral lower extremities. Deep tendon reflexes revealed normal for knee jerk and Achilles tendon reflexes bilaterally. Ataxia, cranial nerves, mini mental, and the rest of the neurological examination showed no abnormalities. Preoperative assessments included electrocardiogram, echocardiogram, and chest radiography. Preoperative laboratory assessment was conducted, including routine laboratory tests (electrolytes, liver and kidney function tests, complete blood count), and tumor markers. The results of the laboratory studies were almost within normal range. Spinal CT and MRI were ordered to visualize the spinal lesions, assess the stability of the vertebral column, and to aid in the formulation of a surgical approach. MRI of the spine showed the density of soft tissue, obvious bony destruction in the T12 and L3, and significant spinal cord compression secondary to the intraspinal mass, with increased marrow infiltration of the vertebra (Fig. 4A–D). Tumor infiltrated through the vertebral bodies into the posterior elements, thus extraosseously spread into the lateral aspects of the epidural space extending posteriorly, resulting in severe spinal cord compression (Fig. 5A–J). Spinal CT showed significant bony destruction of T12 and L3 vertebral bodies, highly suggesting malignant spinal tumors (Fig. 6A–H). The bone scanning revealed high intake in the T12 and L3, with high suspicion of spinal metastases (Fig. 7).

**Figure 8:**
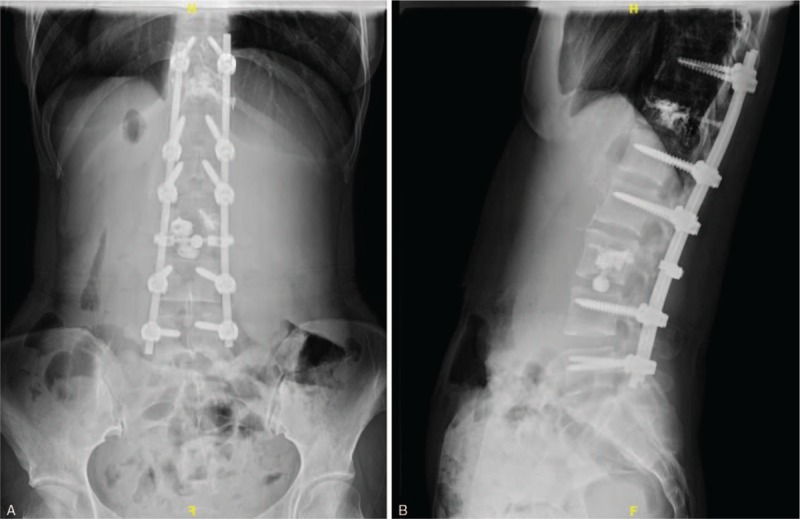
(A,B) Posteroanterior (PA) and lateral x-ray images of the lumbar spine obtained postoperatively.

**Figure 9:**
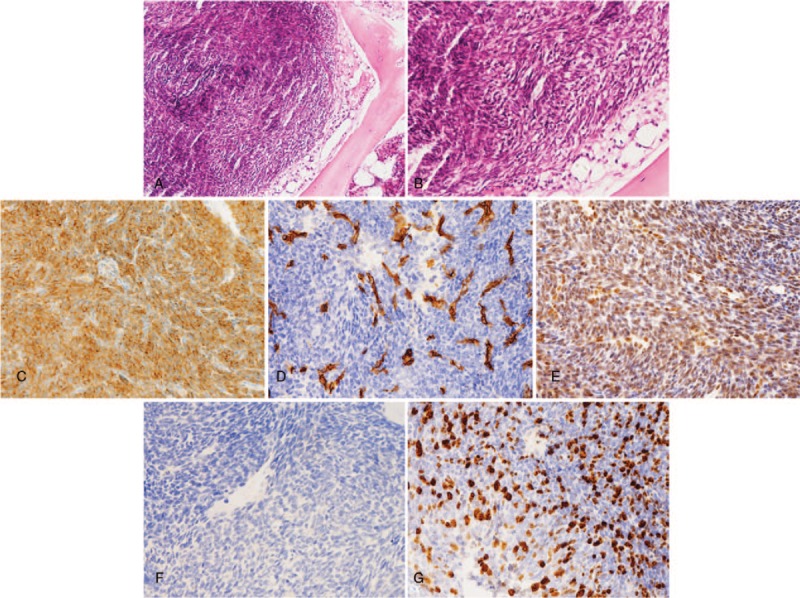
Pathologic histology of spinal metastases. (A,B) Microphotography showing characteristic nests of tumor cells separated by vascular septa (Zellballen) with cells showing significant nuclear pleomorphism with prominent nucleoli (H&E, original magnification 100× and 200×). (C) Bcl-2 immunostaining is strongly positive in the tumor cells. (D), CD34 immunostaining shows strong, diffuse cytoplasmic staining in the tumor cells. (E) The sustentacular cells of the spinal metastases showing characteristic staining of FL1. (F) S100 immunostaining is positive. (G) Ki-67 immunostaining shows 40% Ki-67 positive cells. Ki-67 staining is localized in the tumor nuclei.

Subsequently, posterior spinal canal decompression, complete resection of T12 and L3 lesions, internal fixation from T11 to L5, and cement augmentation of T12 and L3 were performed to destroy the metastatic spinal tumor and stabilize the spine. After general anesthesia induction and intubation, the patient was placed in a prone position for dorsal access to the lumbar spine. For the posterior approach, the paraspinal muscles were detached gently on each side after a midline longitudinal incision was made over the spinous processes. The pedicle entry points were exposed by step-by-step bilateral dissection. In bilateral T11, L1, L2, L4, L5 pedicles, the screw paths were prepared routinely and the pedicle screws were inserted. During the operation, lesions were found in the left part of the lamina and pedicle of L3. The spinous processes and interspinous ligaments of T12, L2, and L3 were removed, bilateral laminae of T12 and L3 were bitten, as well as the lower half of the lamina of L2. After the ligamentum flavum was bitten from the spinal canal, lesions invading the T12 vertebral body and its posterior wall and dark red lesions in the epidural space of T12 and L3 were seen clearly, and the dural sac was significantly compressed. Tumor tissue in spinal canal was completely bitten, and tumor tissues in lateral wall and pedicle of L3 vertebral body and in posterior wall of T12 were scraped. The examination showed that the dural sac and nerve root had been fully released, thus the dural sac pulsed well and the decompression was satisfactory. The wound was immersed in 300 ml distilled water with 50 mg cisplatin for 10 minutes, then washed with a large amount of physiological saline. Then, under C-arm fluoroscopy, the lesions were drilled through special sleeves of T12 and L3 pedicle puncture for vertebroplasty, and the resected tissues were sent for pathological examination. Then the special bone cement for vertebroplasty was modulated. Because the patient did not exhibit hemodynamic instability to the placement of the pedicle screws, bone cement was filled with bone defect after excision of T12 and L3 vertebral bodies. Under fluoroscopy, 6.4 ml and 8.0 ml of bone cement for augmentation were pushed into T12 and L3 vertebral bodies respectively, and fixation using a screw-rod system was employed. Visual inspection using the intraoperative fluoroscopy showed optimal position of all pedicle screws and the dispersed position of bone cement was also good. The incision was closed. Intraoperative blood loss was approximately 500 mL, thus we used erythrocyte 4 U and plasma 200 ml. Postoperatively, the patient was referred to the ICU and transferred to general ward the next day. An x-ray after the surgery confirmed the correct positioning of the implants and bone cement and no signs of displacement of the screws and rods (Fig. 8A and B). The postoperative pathology report confirmed metastatic mesenchymal chondrosarcoma to the spine. Pathological result was positive for Bcl-2, CD34, FL1, S100, with 40% Ki-67 positive nuclei (Fig. 9A–G). Thus, the patient experienced pain relief and improvement of leg numbness. The patient was unwilling to undertake any further treatments and was discharged and monitored on an outpatient basis.

One week after the spinal surgery, the patient's muscle strength of bilateral lower extremities improved to grade V compared to the preoperative status, grade V-, and the sensation to pin-prick and fine-touch of bilateral lower limbs returned to normal. Moreover, VAS score of her back pain improved to 0-1 points compared to the preoperative status, 7 to 8 points. Postoperatively, the patient underwent rehabilitation therapy and was discharged and monitored as an outpatient. The postoperative 1-year follow-up visit showed no tumor progression and no new symptoms. There were no complications associated with the spinal surgery during the follow-up period.

## 3 Discussion

Chondrosarcoma (CS) is a cartilage-forming, low-grade malignant neoplasm that accounts for approximately 10% of all bone tumors, with less than 10% of CS involving the spine.^[1–3]^ Few reports of primary or metastatic CS involving the spinal region causing clinical symptoms have been documented so far, thus there is yet a consensus on the treatment for CS in the spine.^[4,5]^ Mesenchymal chondrosarcoma (MCS) is a malignant tumor arising from bone or soft tissues, whose incidence accounts for 0.2% to 0.7% of malignant bone tumors and 3% to 10% of all chondrosarcomas.^[1–3]^ Patients with intracranial MCS usually present with raised intracranial pressure or cranial nerve palsies. The histological variants can be divided into 3 subtypes: hyaline, myxoid, or mixed.^[4–7]^ For these rare entities, the reported prognosis is variable among different studies. Metastases from intracranial chondrosarcoma to the spine are rarely described in literature and are usually low-grade tumors. The exact incidence of MCS arising from the cranium is unknown to date, but it may be as low as 0.16% of all intracranial tumors.^[1–3]^ For metastatic spinal MCS, typical manifestations include back pain, radiating pain of lower limb, paresthesia, numbness, dysfunction, paraplegia, or incomplete paralysis.^[1–3,8,9]^ Among the above symptoms, paroxysmal back pain can often mimic the most common result of other disorders, making timely diagnosis of metastatic spinal MCS difficult without a high level of suspicion.^[8,9]^ Metastatic spinal MCS can occur at any level along the spinal axis, although they most commonly present in the thoracic region, often presenting with symptoms of back pain, and spinal cord or nerve root compression.^[8–10]^ The location of the spinal lesion determines the neurological deficits, and there is a great deal of variability.

Radiographically, the tumors are often lytic lesions, presenting with cortical bone destruction. Although imaging examinations like CT or MRI scan, which are already the common choices, can provide some valuable evidence for accurate diagnosis of this tumor, though there have not been distinct radiological features that can help to distinguish MCS from other spinal tumors. Radiographs usually give an appearance of soft tissue mass or osteolytic, ill-defined lesion, or well-defined borders with sclerosis.^[4–7]^ The tumor generally shows a lobulated mass with distinct edges and appears isointense on T1WI and T2WI images, with marked homogeneous enhancement. In our reported case, we can see an inhomogeneous density and less-defined mass with a large amount of bony destruction, showing isointense on T1WI and T2WI images in the MRI, which is quite consistent with the common situations. However, due to the unreliability of imaging examination to identify metastatic spinal MCS, clinicians should also consider the hemangioblastoma, neurofibroma, and schwannoma while making a differential diagnosis. The metastatic spinal MCS is commonly a hard or fish-meat like, grayish-white or grayish-red mass, mixed with calcifications sometimes.^[1–4,6,10]^ Through CT or MRI, we can clearly see the margin of the tumor and decide how to remove the tumor via surgical treatment. Clinical studies looking at metastatic spinal MCS is lacking due to the extremely low incidence rate. Imaging studies including CT, MRI, bone scan, and PET/CT are non-specific, making it difficult to differentiate metastatic spinal MCS from other common spinal disorders.^[11–13]^ However, imaging studies may play a crucial role in the decision making of surgical intervention. The “gold-standard” diagnosis of metastatic spinal MCS relies on pathological findings.^[14,15]^ Under light microscope, the tumor has a bi-directional differential feature which consists of undifferentiated small-round mesenchymal cells and islands of hyaline cartilage.^[14,15]^ Immunohistochemistry of biomarkers such as Vimentin, S-100, CK, CD99, NSE, CD56, CD34, and FL1 may help us to differentiate this entity from other spinal tumors.^[14,15]^

Surgery is the best treatment for metastatic spinal MCS causing back pain, radiculopathy, and paralysis.^[16–18]^ This protocol enables accomplishment of 2 objectives: it alleviates the neurological deficits by decompressing the stenosis while provides histopathological specimens for diagnosis at the same time.^[19]^ Nevertheless, there are several considerations to be kept in mind when deliberating on surgical intervention to MCS with spinal involvement, including preoperative spinal instability and selection of operative procedures, possible incomplete tumor resection, intraoperative blood loss and protecting the nerves and vessels, as well as postoperative adjuvant therapy.^[19–22]^

To date, surgical management of metastatic spinal MCS has remained under evaluation, with no standard criteria. No systematic review comparing patient outcomes and surgery types in metastatic spinal MCS has been conducted based on our literature review. Extent of surgical resection is reported to be correlated with overall survival benefit, and en bloc tumor resection with spinal stabilization is the gold standard of surgical treatment. Osteoplasty by cement augmentation may also be a treatment option for patients with metastatic spinal MCS in the spine, who cannot undergo appropriate surgery or decline open surgery.^[20,21]^ However, we need to fully recognize the potential risk of complications in bone cement applications. The safety of this approach still needs to be confirmed in further studies with larger sample sizes and longer follow-up periods. One postoperative complication was cement leakage into the canal and subsequent spinal cord compression.^[20–22]^ Under this circumstance, surgical extent, cement volume, and postoperative complications are critical factors that need further investigation.^[20–22]^

The survival benefit of resection of metastatic spinal MCS is still unproven. However, such a procedure does have the benefit aiming at controlling residual tumor.^[1–3,23,24]^ The improved survival benefited from reducing the tumor burden, decompressing the spinal stenosis to alleviate radiculopathy, and facilitating subsequent chemotherapy and radiation therapy.^[25–27]^ Due to its rarity, the chemotherapy and radiotherapy regimes have not reached a consensus. A main feature of spinal CS is not sensitive to both chemotherapy and radiotherapy, thus patients suffering from metastatic spinal MCS with complex conditions and advanced stages may receive chemotherapy and radiotherapy.^[28–31]^ Moreover, recurrence and metastasis are common postoperative complications due to its invasive nature, which we are anticipating may occur in our patient. They account for a significant percentage of morbidity following resection of metastatic MCS in the spine.^[13,17,30,31,32,33,34]^

## 4 Conclusion

In conclusion, we expect that this case report to educate the clinicians on how we diagnosed and managed a patient with metastatic spinal MCS. Although uncommon, metastatic spinal MCS should be part of the differential diagnosis when the patient presents with back pain and neurological deficits, and pathological examination remain the “gold standard” for diagnosing metastatic spinal MCS. Moreover, osteoplasty by cement augmentation is also a good choice for surgical treatment. However, we need to take the potential risk of complications in bone cement applications into full consideration.^[13,25]^ The management of this unique disorder has yet to be well-documented. With a multidisciplinary team approach, proper planning, and adequate perioperative medical management, metastatic MCS in the spine can be managed much more effectively.

## Acknowledgments

We would like to thank our colleagues at the Department of Orthopaedic Surgery, Peking Union Medical College Hospital, Chinese Academy of Medical Sciences and Peking Union Medical College.

## Author contributions

**Conceptualization:** Shuzhong Liu, Xi Zhou, An Song, Yipeng Wang, Yong Liu.

**Funding acquisition:** Shuzhong Liu, An Song, Yipeng Wang, Yong Liu.

**Investigation:** Shuzhong Liu, Xi Zhou, Yong Liu.

**Resources:** Shuzhong Liu, Xi Zhou, Zhen Huo, Yong Liu.

**Supervision:** Yipeng Wang, Yong Liu.

**Writing – original draft:** Shuzhong Liu, An Song.

**Writing – review & editing:** Shuzhong Liu, An Song, Yipeng Wang, Yong Liu.
